# Enhancer repertoires are reshaped independently of early priming and heterochromatin dynamics during B cell differentiation

**DOI:** 10.1038/ncomms9324

**Published:** 2015-10-19

**Authors:** Mohamed-Amin Choukrallah, Shuang Song, Antonius G. Rolink, Lukas Burger, Patrick Matthias

**Affiliations:** 1 Friedrich Miescher Institute for Biomedical Research, Maulbeerstrasse 66, 4058 Basel, Switzerland; 2 Department of Biomedicine, University of Basel, Mattenstrasse 28, 4058 Basel, Switzerland; 3 Swiss Institute of Bioinformatics, Maulbeerstrasse 66, 4058 Basel, Switzerland; 4 Faculty of Sciences, University of Basel, 4051 Basel, Switzerland

## Abstract

A widely accepted model posits that activation of enhancers during differentiation goes through a priming step prior to lineage commitment. To investigate the chronology of enhancer repertoire establishment during hematopoiesis, we monitored epigenome dynamics during three developmental stages representing hematopoietic stem cells, B-cell progenitors and mature B-cells. We find that only a minority of enhancers primed in stem cells or progenitors become active at later stages. Furthermore, most enhancers active in differentiated cells were not primed in earlier stages. Thus, the enhancer repertoire is reshaped dynamically during B-cell differentiation and enhancer priming in early stages does not appear to be an obligate step for enhancer activation. Furthermore, our data reveal that heterochromatin and Polycomb-mediated silencing have only a minor contribution in shaping enhancer repertoires during cell differentiation. Together, our data revisit the prevalent model about epigenetic reprogramming during hematopoiesis and give insights into the formation of gene regulatory networks.

## Abstract (web summary)

Enhancers in differentiated haematopoietic cells are generally believed to be primed prior to lineage commitment. Here, the authors show that early priming and Polycomb group mediated silencing have minor roles in shaping the enhancer repertoire in differentiated B cells and that most active enhancers are generated *de novo*.

## Graphical abstract

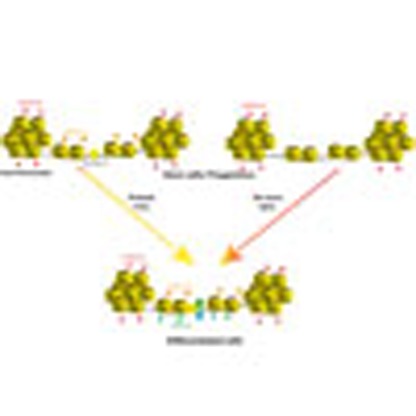

B cells derive from haematopoietic stem cells (HSCs) through multistep differentiation stages. HSCs have both self-renewal and multipotency capacities. They initially give rise to multipotent progenitors (MPPs) that lose self-renewal capacity but keep the ability to generate early progenitors of the lymphoid, myeloid and erythroid lineages. MPPs differentiate into lymphoid-primed MPPs that further give rise to common lymphoid progenitors (CLPs). The CLP compartment contains two distinct populations, all-lymphoid progenitors (ALPs) and B cell-biased lymphoid progenitors (BLPs)^1^. ALPs retain the full lymphoid potential, while BLPs preferentially generate B cells^1^ through multiple stages that are functionally distinct: Pre-pro-B, Pro B, Pre-BI, large and small pre-B II, immature B and finally mature B cells^2^,^3^.

B cell development is controlled by the interplay of a cohort of transcription factors (TFs) and DNA cis-regulatory elements (cis-REs)^4^,^5^,^6^. This interaction is crucial to establish transcriptional programs specific to each differentiation stage. Promoters and enhancers are the two major types of cis-REs in eukaryotes. Enhancers are distal cis-RES that can be located hundreds of kilobases (kb) away of their target genes and play a central role in the activation and fine-tuning of their target promoters^7^. In mammalian cells, enhancer elements have been divided into two major categories, active and primed^8^, that can be distinguished functionally and by specific histone modification patterns. Active enhancers are characterized by the concomitant presence of H3K4me1 together with acetylation marks such as H3K27ac^9^ and are associated with actively transcribed genes, while primed enhancers are solely marked by H3K4me1, lack acetylation marks and their target genes are weakly or not expressed. A subset of primed enhancers are also additionally marked by the Polycomb group (PcG)-related repressive mark H3K27me3; these enhancers, initially identified in human embryonic stem (ES) cells, have been termed poised enhancers^10^. Primed enhancers are thought to be bookmarked for rapid activation in response to environmental or developmental signals.

Cell differentiation from pluripotent stem cells requires not only the activation of specific sets of genes characteristic of the differentiated cell phenotype but also efficient and temporally controlled silencing of pluripotency and lineage inappropriate genes. The main chromatin-associated repressive mechanisms are the PcG-mediated repression and heterochromatin. PcG targets harbour the H3K27me3 mark, which is catalysed by EZH1 and 2 enzymes, two methyl-transferases belonging to the PRC2 complex^11^,^12^. Heterochromatin-enriched loci are marked by H3K9me2/3, a reaction catalysed by the H3K9 methyl-transferases G9A and G9a-like protein^13^. It has been reported that ES cells possess less expanded heterochromatin blocks than differentiated cells^14^,^15^,^16^. These observations suggest that the reduced prevalence of heterochromatin in stem cells plays a role in their developmental plasticity. However, this model was challenged by another study showing that the distribution of heterochromatin is largely conserved between ES cells and differentiated neurons^17^. The dynamics of heterochromatin in adult stem cells and their progeny have been less studied. Furthermore, the crosstalk between heterochromatin and the PcG machinery is a matter of debate: although some reports showed that these two mechanisms are mutually exclusive^17^, other studies proposed that they can cooperate to exert their silencing function^18^.

Although the epigenetic profiles at specific B cell stages are well described^19^, transitions between them have been little investigated. So far it is unclear how the features of enhancers change during the transition from multipotent stem cells to committed progenitors and then to differentiated cells such as mature B cells. The prevailing model is that the enhancer landscape is largely established in early haematopoietic progenitors and that multilineage priming of enhancer elements precedes commitment to the lymphoid or myeloid lineages. This model implies that enhancers used in terminally differentiated cells are pre-marked by H3K4me1 (that is, primed) in early stages before their activation during differentiation or in response to stimuli^20^,^21^,^22^. This model was recently challenged by investigations in the myeloid system, which found only limited enhancer priming in early myeloid progenitors^23^. The role of early enhancer priming during B cell differentiation, before and after the lineage commitment, has not been thoroughly investigated. Furthermore, the role of repressive epigenetic mechanisms in reshaping enhancer repertoires is poorly understood.

Here, we use a genome-wide chromatin immunoprecipitation (ChIP)-sequencing approach to investigate the enhancer dynamics in three developmental stages of the murine haematopoietic system. To this end, we compare the profiles of H3K4me3, H3K4me1, H3K27ac, H3K27me3 and H3K9me2 in uncommitted progenitors/HSCs, committed pro-B and splenic mature B cells. In contrast to the prevalent model, we find that early priming has only a minor contribution in building the enhancer repertoire utilized in terminally differentiated cells. Rather, we find the enhancer landscape to be dynamically reshaped at each step of differentiation. The changes involve establishment of novel enhancers as well as closing and reopening of pre-existing ones. Globally, we find that the vast majority of enhancers are *de novo* established in the cell stages where they are required to control their target genes, without prior priming in earlier stages. Furthermore, the analysis of H3K9me2 reveals that the distribution of heterochromatin is largely invariant from HSCs to mature B cells. We also find that the heterochromatin dynamics and PcG-mediated silencing play only minor roles in reshaping enhancer repertoires during B cell differentiation. Overall, our data revisit the current model of epigenome reprogramming during the progression from adult pluripotent stem cells to terminally differentiated cells.

## Results

### Enhancer classification in HSCs and different B cell stages

Our primary goal was to define the enhancer landscape and its dynamics during B cell development. We focused on three major stages: pluripotent progenitors with HSC potential, committed Pro B cells and mature B cells. As pluripotent progenitors we used immortalized haematopoietic progenitors, as previously described^24^,^25^. Briefly, bone marrow (BM) cells were transduced with a retrovirus encoding a NUP98–HOXB4 fusion protein and cultured in SCF/IL6 containing media. These cells have stem cell properties as they are able to reconstitute all haematopoietic compartments in lethally irradiated mice and can be serially transplanted^24^,^25^; long-term HSCs, short-term HSCs (Supplementary Fig. 1A) and B cells (Supplementary Fig. 1B), among other lineages, can derive from these cells. Thus, the NUP98–HOXB4 transduced haematopoietic progenitors are pluripotent and have stem cell properties and therefore will hereafter be referred to as HSCs. Pro B cells were expanded on feeder cells in the presence of IL-7 and splenic mature B cells were induced to proliferate by LPS.

**Figure 1:**
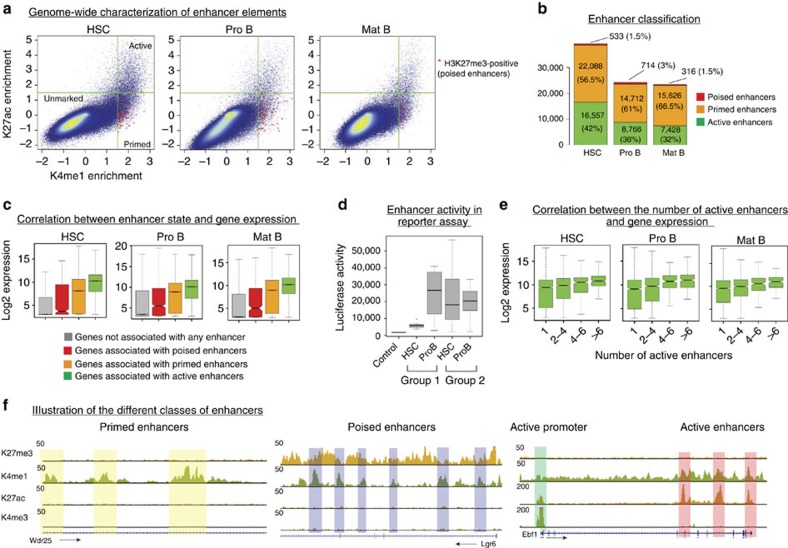
Genome-wide characterization of enhancer elements in HSCs and different B cell stages. (**a**) H3K4me1 versus H3K27ac enrichments (over input) for 1 kb sliding windows across the genome. Regions overlapping with annotated promoters or enriched in H3K4me3 were excluded. Green lines indicate the cut-offs used to select enriched windows for both signals (Materials and methods). Windows additionally enriched for the H3K27me3 mark are marked by red stars (*). (**b**) Putative enhancers (corresponding to merged windows enriched in H3K4me1) were classified according to their enrichment in H3K27ac and H3K27me3 signal. H3K4me1+/H3K27ac+ loci correspond to active enhancers; H3K4me1+/H3K27ac- loci correspond to primed enhancers and H3K4me1+/H3K27me3+ loci to poised enhancers.(**c**) Box plots showing expression levels of genes according to their association with distinct classes of enhancers. (**d**) Functional validation of enhancer states by a reporter assay. Box plots show the luciferase activity for different categories of enhancers in HSCs and Pro B cells. Group 1 consists of enhancers primed in HSCs and active in Pro B cells (n=8); group 2 consists of enhancers active in both HSCs and Pro B cells (n=11). An enhancer-less construct (see Material and methods) was used as a negative control. Raw data are presented in Supplementary Fig. 7D. (**e**) Gene expression levels as a function of the number of associated active enhancers. (**f**). Genome browser snapshots illustrating primed enhancers (*Wdr25* locus), poised enhancers (*Lgr6* locus), and active enhancers and promoter (*Ebf1* locus) in Pro B cells. HSC, haematopoietic stem cells; Mat B, mature B cells; Pro B, progenitor B cells.

To identify enhancer elements, we generated genome-wide maps for the histone modifications H3K4me1 and H3K4me3. These two marks are known to distinguish enhancers from promoters: active promoters are characterized by high level of H3K4me3 and low level of H3K4me1, while enhancers exhibit high H3K4me1 and low H3K4me3 levels^26^. Thus, we identified putative enhancers as loci not overlapping with annotated promoters, lacking H3K4me3 and showing significant enrichment in H3K4me1 signal over the corresponding input chromatin (see Material and methods). Based on these criteria, we identified ∼40,000 putative enhancers in HSCs and 25,000 putative enhancers in Pro B and mature B cells (Fig. 1a,b).

We then classified putative enhancers based on their enrichment in H3K27ac, which marks active enhancers^9^ and H3K27me3, which marks poised enhancers^10^; putative enhancers lacking these two marks and enriched solely in H3K4me1 were considered to be primed enhancers. Based on these criteria, we found that in HSCs roughly 42% of enhancers were active, 56.5% primed and only 1.5% poised. These proportions were moderately different in Pro B (36% active, 61% primed and 3% poised) and mature B cells (32% active, 66.5% primed and 1.5%) (Fig. 1b). We observed a reproducible reduction in the number of enhancers in Pro B and mature B cells relative to HSCs. This reduction was more pronounced at active enhancers, suggesting less enhancer usage in B cells.

### Enhancers act synergistically to promote gene expression

To investigate the relationship between enhancer status and gene expression, we performed RNA-sequencing in all three differentiation stages. We assigned each identified enhancer to the closest promoter, allowing a maximal distance of 500 kb between enhancer and target promoter (see Material and methods). In all three developmental stages genes associated with active enhancers show on average the highest expression levels, followed by genes associated with primed enhancers, poised enhancers and genes not associated with any enhancer (Fig. 1c). These observations were functionally validated by testing a number of putative enhancers in a reporter assay. For this, different categories of enhancers (that is, primed in HSCs and active in Pro B, or active in both stages) were tested for their ability to drive the expression of the nano-luciferase gene (Fig. 1d, Supplementary Fig. 7D, see Material and methods). We found that the putative enhancers identified as being primed in HSCs and active in Pro B cells were indeed significantly more active in Pro B cells than in HSCs, although a significant activity could be measured in HSCs when compared with the enhancers-less construct. In contrast, the enhancers that we had identified as active in both stages were roughly equally active in both differentiation stages and globally show a higher activity than primed enhancers (Fig. 1d).

Interestingly, expression levels generally increase with the number of active enhancers associated with a given gene (Fig. 1e), suggesting that enhancers act synergistically to define the expression level of their target genes.

Active enhancers specific to HSCs, Pro B or mature B cells regulate genes associated with various biological functions. Consistent with the known function of the cell type analysed, HSC enhancers are largely associated with genes involved in cytokines regulation, apoptosis and haematopoietic cell differentiation, whereas genes regulated by Pro B and mature B cell-specific enhancers are associated with lymphocyte differentiation and activation. Many stage-specific enhancers were also found to be associated with genes involved in diseases of the haematopoietic system, such as leukemia or lymphoma (Supplementary Fig. 2).

### Enhancers are shaped by ubiquitous and stage-specific TFs

**Figure 2:**
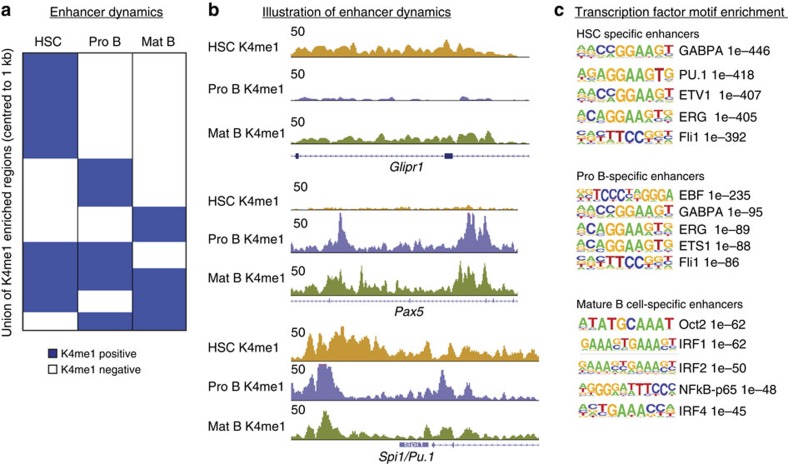
Enhancer repertoires are dynamically reshaped from HSCs to mature B cells. (**a**) Heatmap showing the dynamic behaviour of the H3K4me1 mark in HSCs, Pro B and mature B cells. H3K4me1-positive regions are shown in blue, H3K4me1-negative regions in white. (**b**) Genome browser snapshot illustrating different kinds of cell type-specific enhancers: marked in HSCs and mature B but not in Pro B cells (top, *Glipr1* locus), marked in Pro B and mature B cells but not in HSCs (middle, *Pax5* locus) or marked in all stages (bottom, *Spi1*/*Pu1* locus). (**c**) TF motif enrichments for enhancers specific to HSCs, Pro B or mature B cells. Sequence logos and *P* values are shown for the most highly enriched sequence motifs. Only motifs corresponding to TFs that are expressed in the respective cell type are shown (only ETS1 in HSCs and Oct4 in mature B cells were excluded).

Although enhancer priming is well described for a number of known enhancers in the haematopoietic system^20^, the balance between early priming and *de novo* establishment of new enhancers during differentiation or in response to stimuli has not been rigorously addressed. To examine this, we asked three major questions: (i) how dynamic is the enhancer repertoire from HSCs to fully differentiated cells?; (ii) what is the fraction of primed enhancers in HSCs that become active during differentiation?; and (iii) what is the fraction of active enhancers in differentiated cells that were primed in earlier stages? To answer these questions, we first compared H3K4me1 profiles in HSCs, Pro B and mature B cells. This comparison showed that the enhancer repertoire, as characterized by the H3K4me1 mark, is highly dynamic from HSCs to Pro B cells and from Pro B to mature B cells (Fig. 2a). Although the enhancer repertoires of Pro B and mature B cells are more similar to each other than to the repertoire of HSCs (Supplementary Fig. 3A), dynamic reshaping is observed at all stages of the differentiation process. These changes involve *de novo* establishment of new enhancers that are stage-specific, as well as closing and reopening of pre-existing enhancers (Fig. 2a,b).

The enhancer landscape is largely shaped by the cooperative binding of ubiquitous and cell type-specific TFs^4^,^27^. To address the role of these two categories in enhancer establishment during B cell development, we analysed the identified enhancer sequences for matches to known TF motifs. Enhancers specific to HSCs were largely enriched for PU.1 and many variants of ETS motifs (Fig. 2c). Enhancers specific to Pro B cells were highly enriched for motifs for the B cell-specific TF EBF1 (refs 28, 29) and variants of ETS motifs (Fig. 2c); E2A and PU.1 motifs were also found to be enriched, but with less significant *P* values. Finally, mature B cell-specific enhancers were found to be enriched for binding motifs of Oct2, NFkB and IRF1/2/4, factors known to be important in mature B cells^30^,^31^,^32^. These results clearly support the model that cell type-specific TFs can induce the establishment of new enhancers that were absent in earlier differentiation steps in a stage and context-dependent manner.

### Stage-specific enhancers are rarely primed in early stages

**Figure 3:**
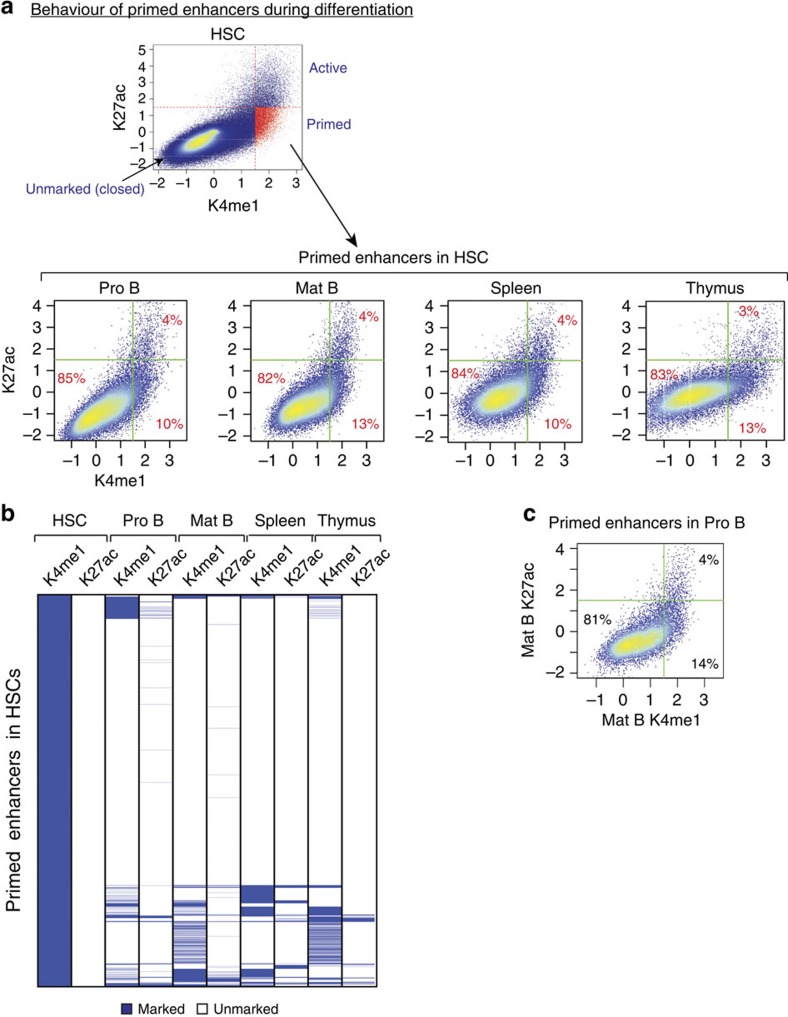
Enhancers primed in HSCs rarely become active at later differentiation stages. (**a**) Chromatin state of enhancers primed in HSCs was investigated in Pro B, mature B cells, spleen and thymus. H3K4me1 and H3K27ac enrichments in the indicated cell types were calculated at genomic coordinates corresponding to primed enhancers in HSCs. Lines indicate cut-offs used to select enriched regions for each signal. The proportions of the different populations are indicated in the scatter plots. (**b**) Heatmap displaying enhancers primed in HSCs and clustered based on their H3K4me1 and H3K27ac enrichment in the indicated cell types and tissues. (**c**) Similar to A, the chromatin state of enhancers primed in Pro B cells was investigated in mature B cells.

We next asked what fraction of primed enhancers (K4me1^+^/K27ac^-^) in HSCs become active (K4me1^+^/K27ac^+^) in differentiated cells. To this end, we selected all primed HSC enhancers and calculated their H3K27ac and H3K4me1 enrichments in Pro B or mature B cells, as well as in spleen and thymus using publicly available data^33^. Surprisingly, we found that only a very small fraction of primed enhancers (4%) in HSCs become active in Pro B or mature B cells (Fig. 3a,b). A small fraction of primed enhancers in HSCs remain primed in Pro B and mature B cells, whereas the majority of them (∼80%) lose K4me1 and become closed in Pro and mature B cells. Similar observations were made for spleen and thymus.

Furthermore, we found that only a small fraction of primed enhancers in Pro B cells become active in mature B cells, while the majority lose both K4me1 and K27ac (Fig. 3c).

We also investigated the behaviour of poised enhancers in subsequent stages. Similar to primed enhancers, the few poised enhancers identified in HSCs and Pro B cells rarely become active in later stages (Supplementary Fig. 4A,B).

**Figure 4:**
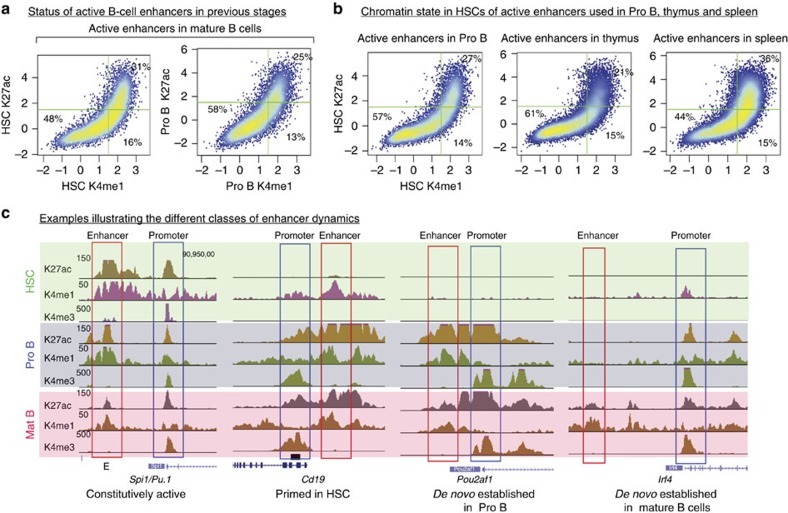
Most enhancers active in differentiated cells are not primed in stem cells or progenitor stages. (**a**) Chromatin state of active enhancers in mature B cells was investigated in HSCs and Pro B cells. Scatter plots display H3K4me1 and H3K27ac enrichment for HSCs and Pro B cells at the coordinates of active enhancers in mature B cells. The percentage of enhancers in the respective quadrants are indicated. (**b**) Similar to A, the chromatin state of active enhancers in Pro B cells, spleen and thymus was investigated in HSCs. (**c**) Genome browser snapshot illustrating the different classes of enhancer dynamics.

Consistent with this result, we found that only a minority of active enhancers in mature B cells were primed in HSCs or in Pro B cells, while the majority were either already active in earlier stages or completely closed (Fig. 4a). To exclude the possibility that LPS activation may affect enhancer usage in mature B cells, we also generated H3K27ac maps in splenic resting B cells. We found only a modest reduction in the overall number of enriched regions compared with LPS-induced cells (Supplementary Fig. 5A). This is consistent with a previous study showing that in resting B cells the level of histone acetylation as measured by H3K9ac mapping is lower than in other cell types, such as in proliferating *Rag2*−/− Pro B cells^19^. However, in spite of this slight reduction in the number of enriched regions, the H3K27ac signal is globally similar between LPS-induced and resting B cells (Supplementary Fig. 5A). Furthermore, using the H3K27ac data of resting B cells to identify active enhancers and interrogating their status in Pro B cells and HSCs leads to similar results as for LPS-induced B cells (Supplementary Fig. 5B).

We also considered the possibility that enhancers used in B cell lineages might be primed in an intermediate differentiation stage such as the CLP stage. We therefore used publicly available data sets generated in CLPs^22^ and compared how H3K4me1 and H3K27ac change between CLPs, Pro B and mature B cells. The results presented in Supplementary Fig. 5C demonstrate that, in this case also, only a small fraction of the enhancers active in Pro B and mature B cells were primed in CLPs.

Similar observations were also made for active enhancers identified in spleen and thymus when investigated for their status in HSCs (Fig. 4b). Taken together, these results challenge the prevalent model and indicate that early priming events represent an exception rather than the rule: the majority of active enhancers in terminally differentiated cells examined here are *de novo* established in the stage where they are required, with limited priming in previous stages. Fig. 4c presents genome browser views illustrating the different combinations of enhancer dynamics observed from HSCs to mature B cells.

### Enhancer usage discriminates close differentiation stages

**Figure 5:**
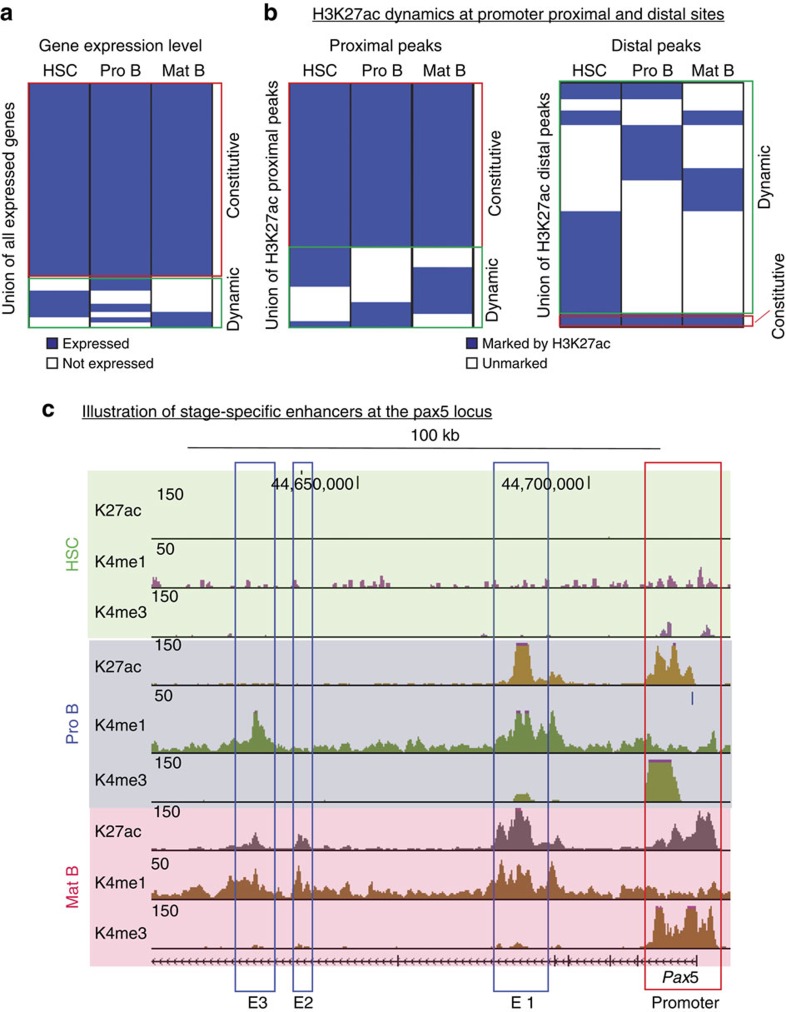
Enhancers are used in a stage-specific manner. (**a**) Heatmap showing gene expression levels dynamics in HSCs, Pro B and mature B cells. Genes were divided into two categories, expressed (blue) and not expressed (white). (**b**) Heatmap showing H3K27ac signal dynamics at promoters (proximal peaks, left panel) and outside of promoters (distal peaks, right panel). H3K27ac-positive regions are shown in blue and H3K27ac-negative regions in white. (**c**) Browser snapshot illustrating stage-specific enhancer usage for the *Pax5* gene in Pro B and mature B cells. E1, E2 and E3 stand for Enhancers 1, 2 and 3. Enhancer 1 is common to Pro B and mature B cells, enhancer 2 is specific to mature B cells and enhancer 3 is solely marked by H3K4me1 in Pro B cells and acquires H3K27ac in mature B cells.

To address the question whether enhancer usage can distinguish closely related cell stages sharing similar gene expression patterns, we compared gene expression levels for HSCs, Pro B and mature B cells. For simplicity, genes were divided into two classes, expressed or not expressed (see Material and methods and Supplementary Fig. 6) and only genes expressed in at least one stage were considered further. Gene expression patterns are similar between the three stages with only a small fraction found to be differentially expressed (Fig. 5a). Consistent with this, H3K27ac shows only a moderate amount of variability at promoter regions (proximal peaks) between HSCs, Pro B and mature B cells (Fig. 5b, left panel). In contrast, distal H3K27ac peaks (that is, active enhancers) show distinct cell stage-specific patterns (Fig. 5b, right panel), indicating cell stage-specific usage of enhancer elements in cell types sharing highly similar gene expression patterns, in accordance with earlier finding in human cells^34^. This specificity is illustrated by enhancers regulating the *Pax5* gene; while in Pro B cells this gene is controlled by a well-studied enhancer located in the fifth intron (E1)^35^, in mature B cells, the *Pax5* gene appears to be associated with two additional enhancers (E2 and E3) located in the sixth intron (Fig. 5c).

### Heterochromatin dynamics during differentiation

**Figure 6:**
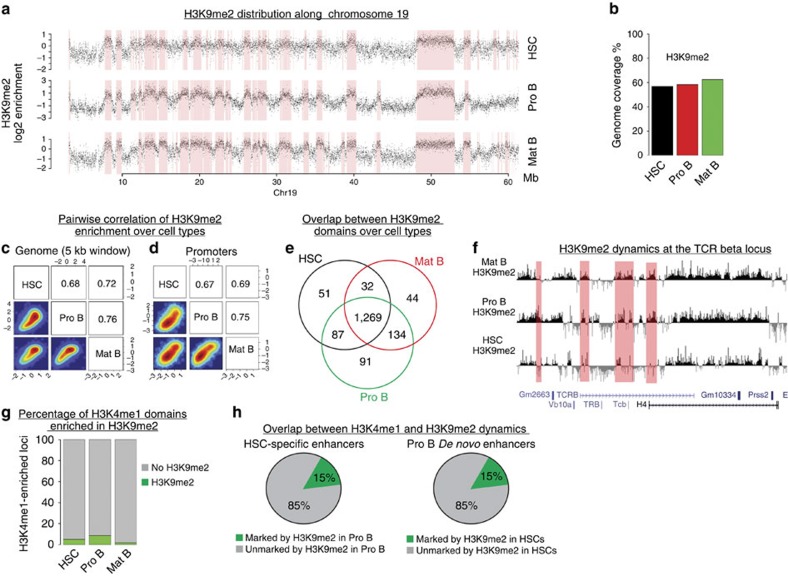
Heterochromatin distribution is largely stable from HSCs to mature B cells. (**a**) H3K9me2 enrichments in 5-kb windows along the entire chromosome 19 (Chr19) in HSCs, Pro B and mature B cells. Identified heterochromatic domains are indicated by pink rectangles. Mb: megabases. (**b**) Percentage of the genome marked by H3K9me2 in HSCs, Pro B and mature B cells. Only genomic windows with at least one read in any of the input or H3K9me2 samples was considered. (**c**) Pairwise comparison of H3K9me2 enrichment in 5 kb sliding windows for HSCs, Pro B and B cells. Pearson correlations are indicated. (**d**) Similar to C, for promoters (± 1 kb around transcription start sites). (**e**) Venn diagrams showing the number of nucleotides (in Mb) that are common between the identified H3K9me2 domains in HSCs, Pro B and mature B cells. (**f**) Browser snapshot illustrating local changes in H3K9me2 at the T-cell receptor (*TCR*) *beta* locus. (**g**) H3K4me1 marked loci were classified according to their overlap with H3K9me2 domains. (**h**) Enhancers specific to HSCs and closed in Pro B cells were investigated for their overlap with H3K9me2 domains in Pro B cells; similarly, *de novo* enhancers generated in Pro B cells and absent in HSCs were investigated for their overlap with H3K9m2 domains in HSCs. The proportions of H3K9me2-marked and unmarked loci are indicated.

To investigate whether heterochromatin plays a role in modulating cis-RE dynamics we generated genome-wide maps for H3K9me2, a histone mark characterizing heterochromatin-enriched loci^15^. Visual inspection of H3K9me2 enrichments along the genome revealed a clear separation into large domains of H3K9me2-depleted and H3K9me2-enriched regions (Fig. 6a). To determine the H3K9me2-enriched domains genome-wide, we used a two-state Hidden Markov Model (HMM, see Material and methods). The identified H3K9me2-enriched heterochromatic domains are shown in red in Fig. 6a for the entire chromosome 19. In all cell types analysed here, the domains cover roughly half of the genome (Fig. 6b). Interestingly, H3K9me2 is largely invariant during differentiation, as evidenced by high correlations at the level of 5 kb tiling windows (Fig. 6c) and promoters (Fig. 6d), as well as by a strong overlap of the HMM-identified domains (Fig. 6e). These findings are in line with previous observations in ES cells and neurons^17^. Despite the global similarity, rare loci show detectable changes in H3K9me2 enrichment (Fig. 6e), for example at the T-cell receptor beta locus, where we observed an overall increase in H3K9me2 signal in Pro B and B cells relative to HSCs (Fig. 6f).

To investigate the relationship between heterochromatin and enhancer dynamics, we monitored the overlap of H3K4me1 and H3K9me2-enriched regions during the differentiation (see Material and methods). As expected, only a small fraction of putative enhancers overlap with H3K9me2-enriched regions in each cell stage (Fig. 6g). Additionally, only a small fraction (∼15%) of enhancers that are closed (that is, lose H3K4me1) during the transition from HSCs to Pro B cells become marked by H3K9me2 in Pro B cells (Fig. 6h). Conversely, we found that only∼15% of *de novo* enhancers generated in Pro B cells were marked by H3K9me2 in HSCs (Fig. 6h), indicating that the dynamics of enhancers are largely unrelated to the dynamics of heterochromatin.

## Discussion

The development of terminally differentiated cells from pluripotent stem cells is a stepwise process controlled by the complex interaction between TFs and DNA cis-REs embedded in chromatin. Here, we investigated the dynamic changes in the epigenome landscape during B cell development from HSCs. Our study addressed several aspects of epigenetic regulation, ranging from enhancer dynamics to repression mechanisms controlling gene expression. We focused on three main developmental stages: uncommitted MPPs (HSCs), committed progenitors (Pro B) and terminally differentiated cells (mature B cells). We also took advantage of publicly available data generated in other developmental stages or haematopoietic tissues to perform a multilineage comparison.

Several previous studies suggested that enhancers can be activated in a stepwise manner during development^22^,^35^,^36^. This activation process involves a primed state that corresponds to a pre-marking of enhancers in early stem cell or progenitor stages before their usage in further differentiated stages. Primed and active states can be discriminated by distinct chromatin signatures: primed enhancers harbour H3K4me1 only, whereas active enhancers harbour acetylation marks such H3K27ac and H3K9ac in addition to H3K4me1. In addition to these two major enhancer states, it was also reported that some enhancers can harbour H3K4me1 and the repressive mark H3K27me3; these enhancers were termed poised enhancers and described primarily in human ES cells^10^.

Two very recent studies investigating enhancer dynamics in myeloid cells came to divergent conclusions. Lara-Astiaso *et al*.^22^ found that enhancers used in terminally differentiated myeloid cells are not primed in HSCs, but rather in early myeloid progenitors prior to the execution of the RNA expression program. In contrast, Luyten *et al*.^23^ concluded that meyloid enhancers are generated *de novo* with only limited priming in earlier stages. These divergent conclusions might be due to the usage of different criteria to select enriched regions for histone marks or to the use of slightly different cell populations.

Although enhancer priming in early stages before their activation was demonstrated for several well-studied enhancers in the haematopoietic system^35^,^36^,^37^, the balance in enhancer usage between pre-existing enhancers and *de novo* enhancers had not been addressed genome-wide during B cell differentiation, before and after commitment. Our data indicate that the majority of active enhancers in differentiated cells are not primed in earlier stages. Indeed, we found that most active enhancers in mature B cells are either already active in the previous stages analysed (Pro B and HSCs) or completely closed (unmarked) in these stages. Furthermore, a differential analysis of H3K4me1 and H3K27ac changes between B cells populations and CLPs stage (public data)^22^ indicates that enhancer priming of B cell enhancers is also rare at the CLP stage (Supplementary Fig. 5C). This finding is consistent with the fact that the majority of primed enhancers in HSCs and Pro B cells were not found to become active (that is, positive for H3K27ac mark) in mature B cells. These observations indicate that early priming in previous stages has only a minor contribution to enhancer repertoire establishment during B cell development. Repeating our analysis in the spleen and thymus leads to similar results. This suggests that the minor contribution of priming is a general phenomenon in the haematopoietic lineage and that during cell differentiation from stem cells and progenitors enhancers are dynamically *de novo* generated, with little use of the pre-existing primed enhancer landscape.

Consistent with previous studies, our data also highlight the role of stage-specific TFs in building stage-specific enhancers during B cell development^4^,^5^. Interestingly, closely related cell stages sharing a highly similar gene expression pattern such as Pro B and mature B cells, have nevertheless highly divergent enhancer repertoires. This suggests cell stage-specific enhancer usage to regulate the same set of genes. Furthermore, our analysis indicates that enhancer patterns may be better descriptors of cellular differentiation stages than commonly used gene expression profiles, and may allow for higher granularity.

The abundance of primed enhancers is thought to reflect cell plasticity of stem cells and early progenitors. Our results challenge this dogma as we found similar proportions of primed enhancers in HSCs, progenitors (Pro B) and mature B cells. Although we have deciphered the usage of primed enhancers in HSCs and Pro B cells during B cell development, it is unclear what the functional role of the H3K4me1 marking in the absence of acetylation (indicative of enhancer activity) is. One possibility is that H3K4me1 deposition in some condition can correspond to ‘sterile' binding of TFs, which is not sufficient to induce enhancer activity because of the absence of other binding partners. It is also possible that H3K4me1 represents an epigenetic memory of previous activation events at earlier developmental stages. Nevertheless, we observed that genes associated with primed enhancers show overall a slightly higher expression level than genes not associated with any enhancer, irrespective of the developmental stage considered. Moreover, when these primed enhancers were tested functionally in cell transfection assays, a low but significant activity was observed. This suggests that primed enhancers may already have some weak transcription activation potential. Although we have observed a minor contribution of enhancer priming in earlier stages in building enhancer repertoires in subsequent stages, we cannot exclude the possibility that enhancer priming can occur in a short time window before enhancer activation.

Cell differentiation progresses by repressing pluripotency genes as well as lineage inappropriate genes. Gene silencing is linked to distinct epigenetic mechanisms including DNA methylation, heterochromatin and PcG-mediated repression. In the present study, we focused on the main chromatin-related repression machineries that are heterochromatin and PcG. The role of hetrochromatin prevalence in stem cell plasticity is a matter of debate; although some studies suggest that heterochromatin is more abundant in lineage-restricted cells than in ES cells^14^, other investigations showed that heterochromatin prevalence does not discriminate ES cells from neurons^17^. Heterochromatin prevalence and dynamics in adult stem cells, such as HSCs, and their progeny has been poorly investigated so far. We have used H3K9me2 mapping to interrogate the heterochromatin distribution in HSCs, Pro B and mature B cells. Our data indicate that the distribution of heterochromatin is largely invariant between HSCs and lineage-restricted Pro B and mature B cells. Furthermore, we found that heterochromatin dynamics play only a minor role in shaping the enhancer repertoires during B cell differentiation from HSCs.

Overall our study revisits the prevalent models and provides novel insights into the dynamic regulation of the epigenome landscape during haematopoiesis.

## Methods

### Cells

Nup-Hoxb4 HSCs were generated as previously described in (ref. 24) (see Results section). Pro B cells were sorted as described previously^38^ from the BM of 4–5 weeks old mice with the following markers (ckit+, B220+, CD19−, CD25−, IgM−). Pro B cells were expanded in the presence of IL7 and OP9 feeder cells for 1 week. Mature B cells were sorted from the spleen of 8–10-weeks-old mice as CD19-positive cells using MACS kit and CD19 beads (#130-052-201). Mature B cells were cultured in the presence of LPS for 2 days. C57BL/6 mice were housed in a controlled environment and experiments were conducted in accordance with the ordinance provided by Cantonal Veterinary Office, Basel-Stadt, Switzerland.

### Antibodies

ChIP: H3K4me3 Millipore #07-473, H3K4me1 abcam #ab8895, H3K27me3 upstate # 07-449, H3K27ac Active Motif #39133, H3K9me2 abcam #1220.

FACS: Anti-Sca1-PerCP Biolegend #108122, Anti-c-Kit-APC Pharmingen #553842, Anti-Flt3-PE Ebioscience #17-1171-83, Anti-Lin-Efluor450 Ebioscience #88-7772-72, B220-FITC BD #553087, CD19 ebioscience #Clone: MB19-1.

### Chromatin immunoprecipitation (ChIP)

Cells were fixed with 1% formaldehyde in PBS (20 ml volume/∼1 × 10^8^ cells) for 10 min, followed by quenching with 2.5 M glycine for 5 min. Cells were lysed for 10 min in 1 ml lysis buffer A (10 mM HEPES, pH 8.0, 10 mM EDTA, 0.5 mM EGTA, 0.25% Triton X-100+PI). The supernatant was discarded and the nuclei were incubated for 10 min in buffer B (10 mM HEPES, pH 8.0, 200 mM NaCl, 1 mM EDTA, 0.5 mM EGTA, 0.01% Triton X-100+PI). Next, the nuclei were suspended in chromatin lysis buffer (50 mM Tris-HCl, pH 8.0, 10 mM EDTA, 0.5% SDS) for 30 min. Sonication was performed using Bioruptor Next Gen (Diagenode) at high output intensity for 25 cycles (30 s on/30 s off). Finally the collected supernatant was diluted 5 × with chromatin dilution buffer (250 mM NaCl, 1.67% Triton X-100). The chromatin (25–50 μg) was subjected to the immunoprecipitation with 5 μg of the above mentioned antibodies and incubated overnight. The samples were incubated with either protein A or G coupled to magnetic beads for 1 h. The beads were washed with the following buffers, twice with low salt (0.10% SDS, 1% Triton X-100, 2 mM EDTA, 20 mM Tris pH 8.0, 15 mM NaCl), high salt (0.10% SDS, 1% Triton X-100, 2 mM EDTA, 20 mM Tris pH 8.0, 50 mM NaCl), LiCl (250 mM LiCl, 1 M EDTA, 10 mM Tris pH 8.0, 1% NP-40, 1% Na Deoxychholate) and once with TE (20 mM Tris-HCl, pH8.9, 2 mM EDTA). The protein-DNA complexes were eluted from the beads with 500 μl elution buffer (10 mM Tris-HCl, pH7.5, 1 mM EDTA, 1% SDS, 100 mM NaHCO3) and reverse crosslinked overnight at 65 °C with Proteinase K. ChIPed DNA was isolated by phenol/ chloroform extraction and ethanol precipitated. 10 ng of ChIPed DNA were processed for Illumina high-seq analyser according to the manufacturer's protocol.

### RNA isolation and sequencing

Cells sorted from three different mice were pooled and cultured as described above. Total RNA was extracted using RNAeasy kit from QIAGEN #74104 with on-column DNaseI treatment. 10 ng of RNA were used for library preparation using ScriptSeq v2 RNA-Seq Library Preparation Kit and sequenced according to the Illumina protocol.

### Library preparation for ChIP sequencing

Sequencing libraries were prepared using bar-coded adapters following the standard Illumina library preparation protocol. Four samples carrying different barcodes were pooled at equal molar ratios and subjected to sequencing on Illumina HiSeq 2000 sequencer according to Illumina standards.

### Reporter assays

Loci corresponding to different categories of enhancers were amplified from mouse genomic DNA based on H3K4me1 and H3K27ac peak locations and cloned into pNL1.1 vector (Promega) upstream of the nano-luciferase gene and minimal promoter. 0.4 μg of cloned constructs were electroporated into 2.5 × 10^5^ cells using SF solution (PBC2-00675) and Amaxa 4D-NucleofectorX device. Luciferase activity was assessed 24 h post electroporation using the Nano-Glo Luciferase Assay System #N1110 from Promega and Berthold LB device. A construct containing only the minimal promoter (enhancer-less construct) was used as a negative control. Genomic Coordinates of the cloned sites are provided in Supplementary Table 1

### Computational analyses

Sequencing reads were mapped to the mouse genome (mm9) using the qAlign function, which internally uses Bowtie (http://bowtie-bio.sourceforge.net/), from the QuasR R package^39^ (http://www.bioconductor.org/packages/release/bioc/html/QuasR.html) with default settings. Alignments were shifted by 60 bases, corresponding to an estimated fragment length of 120 bp. For window-based analyses, a sliding window of 1 kb length and a step-size of 0.5 kb was used. For all samples, reads were counted in each window using the qCount function in QuasR (with shift=60) to generate a matrix with read counts for each sample in the defined genomic intervals. The total read counts per sample were normalized to the mean total number of reads across all samples. We calculate the enrichment over input using the following formula (after library-size normalization): (number_of_reads_ChIP+8)/(number_of_reads_input+8). We thus add a pseudo-count of 8 to the tag counts within a given region, which results in an effective reduction of enrichments when the counts are low.

We generated four biological replicates for H3K4me1 in HSCs and Pro B cells and two replicates for this mark in mature B cells (Supplementary Figs 3A and 8). For H3K27ac, we generated two biological replicates per stage (Supplementary Fig. 3B). For H3K27me3, we generated two biological replicates in Pro B and mature B cells and used publicly available data for HSCs generated in the same cellular system used in the current study^24^. For H3K4me3, we generated one replicate per stage and for H3K9me2 two replicates for HSCs and one replicate for Pro B and B cells. Enrichments for sample replicates were averaged. The cut-offs to select regions enriched for H3K27ac and H3K27me3 were set to 1.5 (log2 scale), which separates the two modes of the bimodal distribution of these signals at promoters and thus is a sensible choice for genome-wide classification. For H3K4me3, regions with an enrichment value below 1 (log2 scale) were considered as depleted from this mark and region with enrichment value higher than 3 were considered as positive. The cut-offs for the H3K4me3 mark were estimated from its distribution at promoters where negative and positive population can easily be discriminated. For H3K4me1 several cut-offs were tested, ranging from a log2 enrichment of 1 to a log2 enrichment of 2. Upon visual inspection, a cut-off of 1 (2-fold) was found to be too low for confident detection of enriched regions. We therefore used a more conservative cut-off of 1.5 (∼3 fold). For all marks, overlapping enriched regions were merged to one region.

Peak detection of H3K4me3 and H3K27ac using the MACS software^40^ gave quantitatively similar results to the above described windows-based method.

To determine K9me2-enriched domains, the entire genome was tiled into consecutive windows of 5 kb and for each cell type H3K9me2 enrichments over input were calculated (analogously to the other marks as described above). Only windows with reads in either K9me2 or input samples in any of the three stages were retained (∼95% of all windows). Visual inspection of the enrichments along the genome indicated a clear separation into two classes of broad domains with either positive (K9me2-enriched) or negative (K9me2-depleted) values. To determine these domains genome-wide, we first trained, separately for each cell type, a two-state HMM with Gaussian emission distributions on the enrichment values of chromosome 1 using standard expectation maximization as implemented in the R package mhsmm^41^. As starting values, we used an initial probability of 1 to be in the enriched state, symmetric transition probabilities with 0.9 probability to stay in the same state and 0.1 probability to transit to the opposite state, and Gaussian emission distributions with means −1 and 1 and variances 0.5 and 0.5 for the H3K9me2-depleted and H3K9me2-enriched states, respectively. We then predicted the most-likely domains genome-wide using the Viterbi Algorithm as implemented in the predict function of the mhsmm package.

Promoters were defined as non-overlapping −1 to +1 kb intervals around transcription start sites as defined in the refGene.txt file downloaded from the UCSC genome browser (http://hgdownload.cse.ucsc.edu/goldenPath/mm9/database/refGene.txt.gz, downloaded April 20, 2012). ChIP enrichments at promoters were calculated as described for the windows-based analysis.

TF motif discovery was performed using the HOMER software^4^ using the following command (findMotifsGenome.pl input_list mm9 output_directory -size 1000). The size of the regions used to search for motifs was set to 1 kb. The lists of TF motifs identified were filtered by expression levels.

ChIP sequencing signals were visualized using library-size normalized wig files generated with the qExportWig function (default parameters) from the QuasR package and uploaded as custom tracks to the UCSC Genome browser. Except for H3K9me2, where they represent enrichment over input in 1 kb windows, wig files represent read counts in 100 nt tiling windows of the genome.

Gene expression levels were determined by summing, for each gene, all RNA-seq reads that map to any of the annotated exons of each gene (using the qCount function of QuasR). Annotations were taken from the UCSC refGene.txt file (see above). To compare expression levels between stages, the RNA-seq libraries were normalized to the mean total number of reads and to the average exonic length. The exonic length of each gene was defined as the total number of unique exonic bases stemming from any annotated transcript.

Enhancers were assigned to the closest promoter allowing for a maximum distance of 500 kb. First, active enhancers were assigned to their closest promoters; the promoters associated with active enhancers were excluded from the list of promoters used to assign primed and poised enhancers.

## Additional information

**Accession codes**: All data sets have been deposited in GEO under the accession number GSE60005.

**How to cite this article:** Choukrallah, M.-A. *et al*. Enhancer repertoires are reshaped independently of early priming and heterochromatin dynamics during B cell differentiation. *Nat. Commun.* 6:8324 doi: 10.1038/ncomms9324 (2015).

## Supplementary Material

Supplementary Information Supplementary Figures 1-8 and Supplementary Tables 1-4
