# Supplementary material for: Enhancer repertoires are reshaped independently of early priming and heterochromatin dynamics during B cell differentiation
Source: Nat Commun. 2015 Oct 19;6:8324. doi: 10.1038/ncomms9324 (PMC4633987; doi:10.1038/ncomms9324)
Supplement: Supplementary Information — Supplementary Figures 1-8 and Supplementary Tables 1-4 [file ncomms9324-s1.pdf]

Supplementary Figures

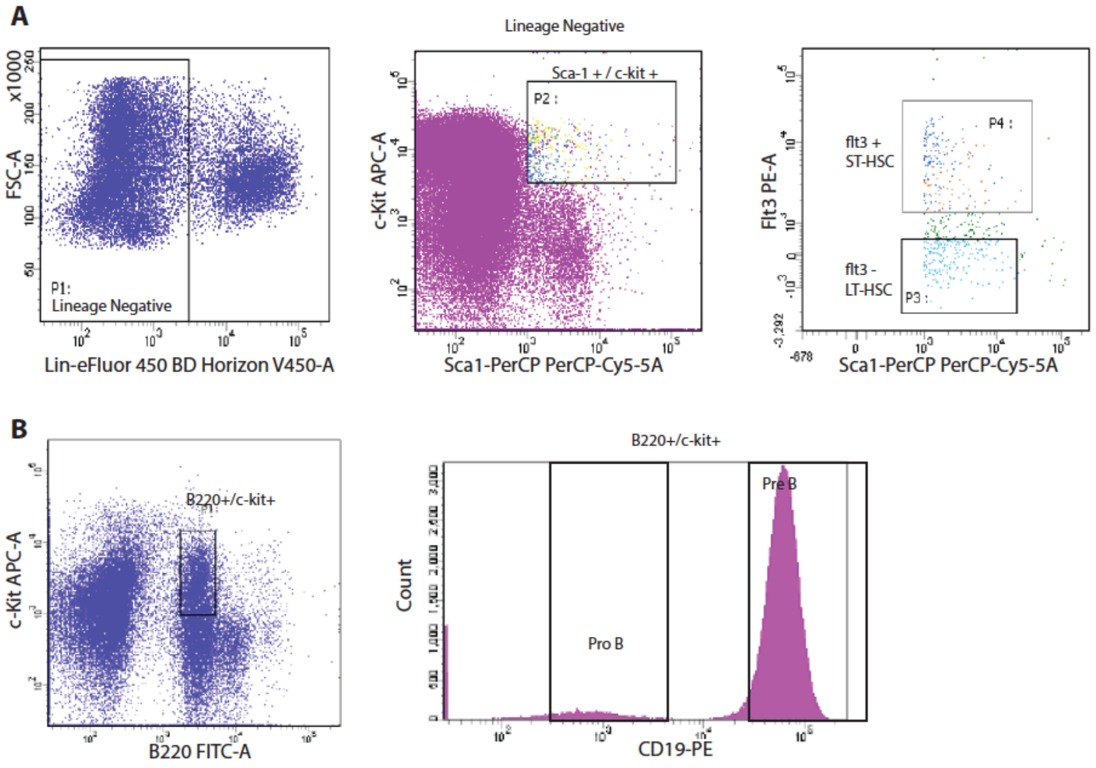

Supplementary Fig. 1: Nup-Hoxb4-HSCs are able to repopulate the hematopoietic system of irradiated Rag2 deficient mice.

A. Nup-hox4-HSCs were injected into irradiated Rag2 deficient mice. After 4 weeks, LT-HSCs (Lineage-, Sca+, c-kit+, flt3-) and ST-HSCs (Lineage-, Sca+, c-kit+, flt3+) were isolated from BM of recipient mice.

B. Pro B cells (B220+, c-kit+, CD19-) and Pre B cells (B220+, c-kit+, CD19+) were also isolated from recipient mice.

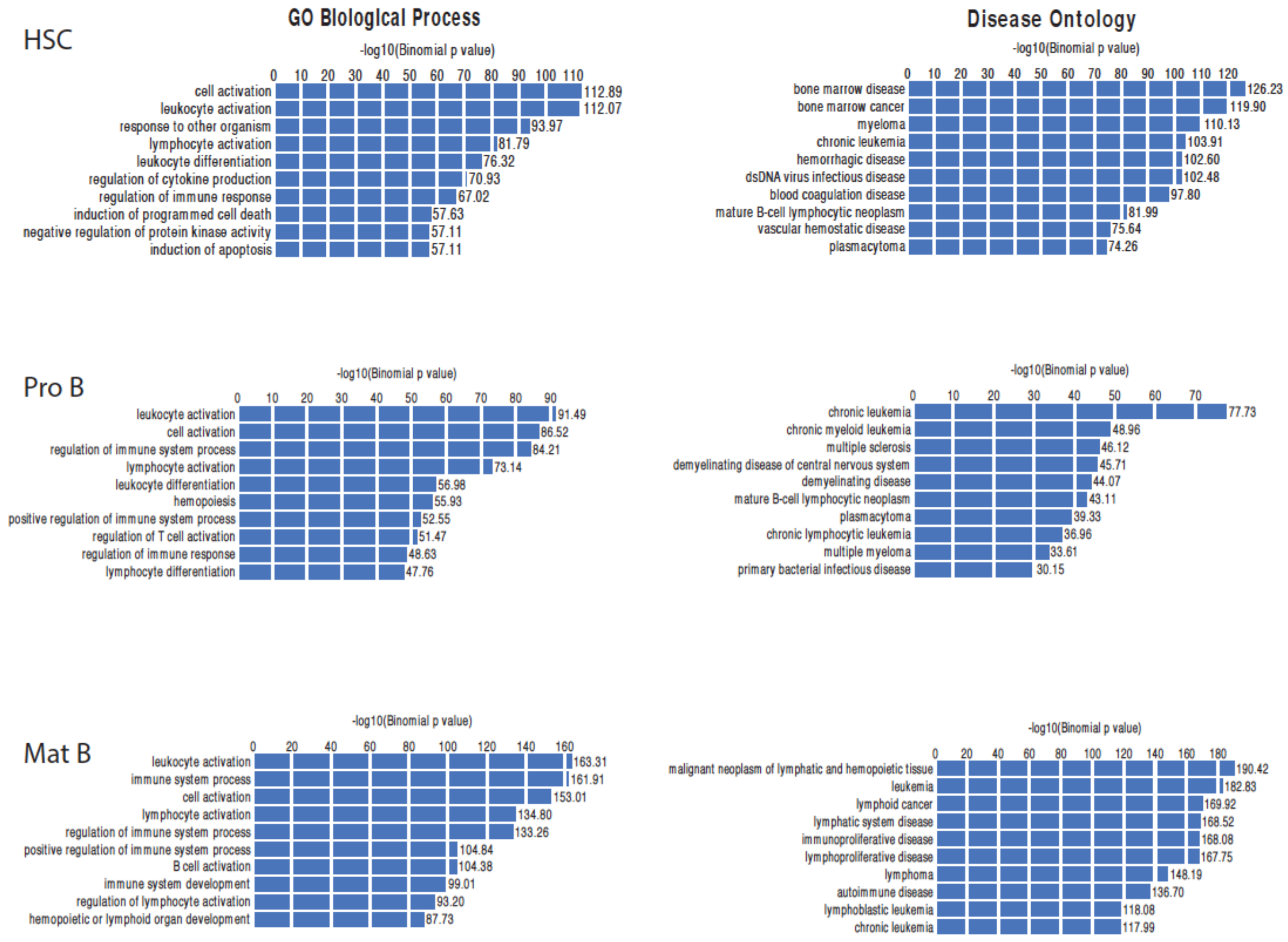

Supplementary Fig. 2: Biological functions and diseases associated with target genes of active enhancers in HSCs, Pro B and mature B cells.

Genomic coordinates of active enhancers were associated to their putative target genes; the resulting set of genes was submitted to gene annotation terms for biological processes and diseases using the Great software (http://bejerano.stanford.edu/) with default settings. Top ten hits and their associated p-values are shown.

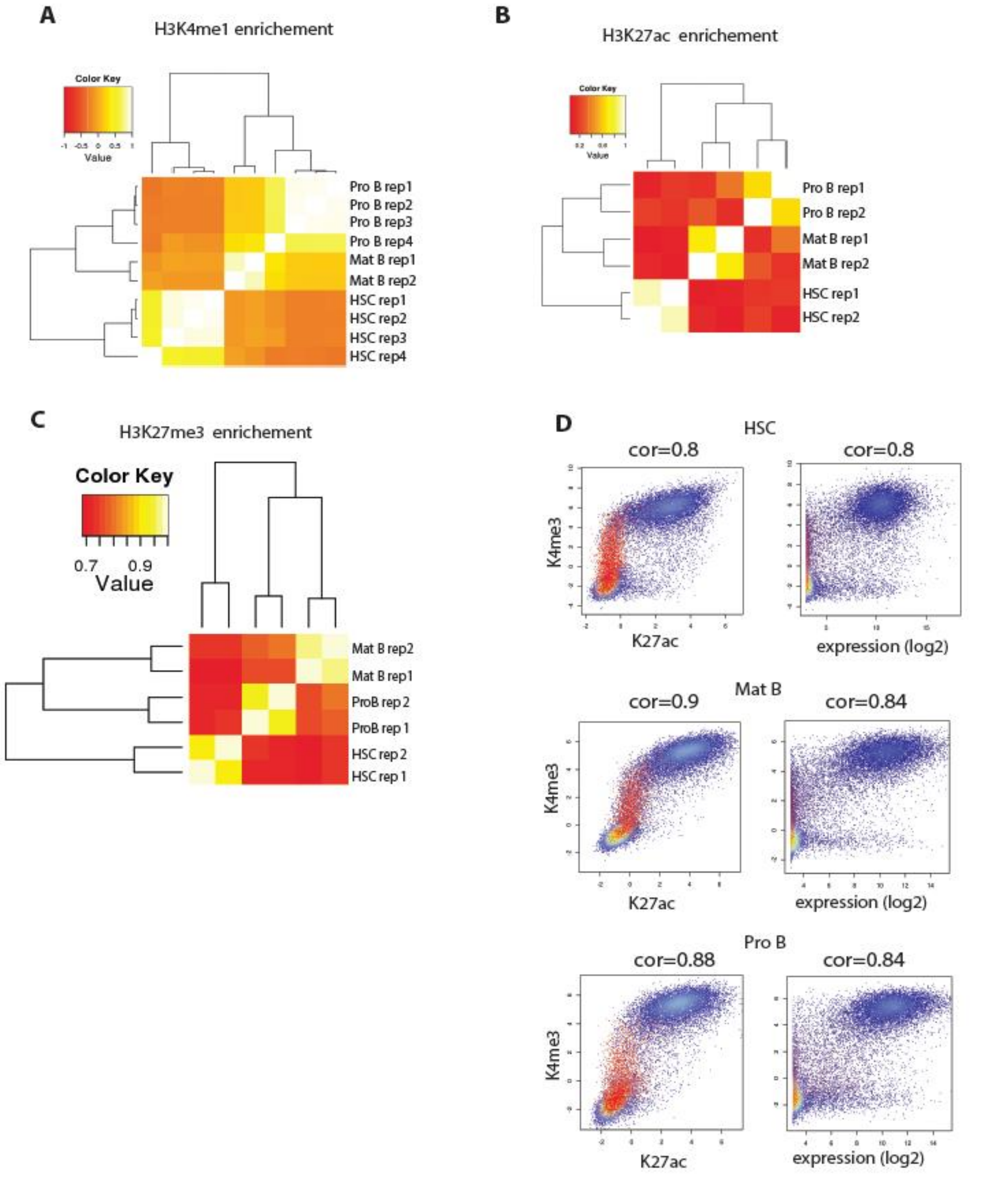

Supplementary Fig. 3: Correlation between replicates across samples.

A and B. Clustering of H3K4me1 (A) and H3K27ac (B) enrichment at enhancer elements based on Pearson correlation. Biological replicates for each stage cluster together.

C. Clustering of H3K27me3 enrichment at promoter regions.

D. Scatter plots of H3K4me3, H3K27ac and RNA sequencing signals at promoter regions. Promoters enriched in H3K27me3 are colored in red. Cor stands for Pearson correlation coefficient.

Behavior of poised enhancers during differentiation

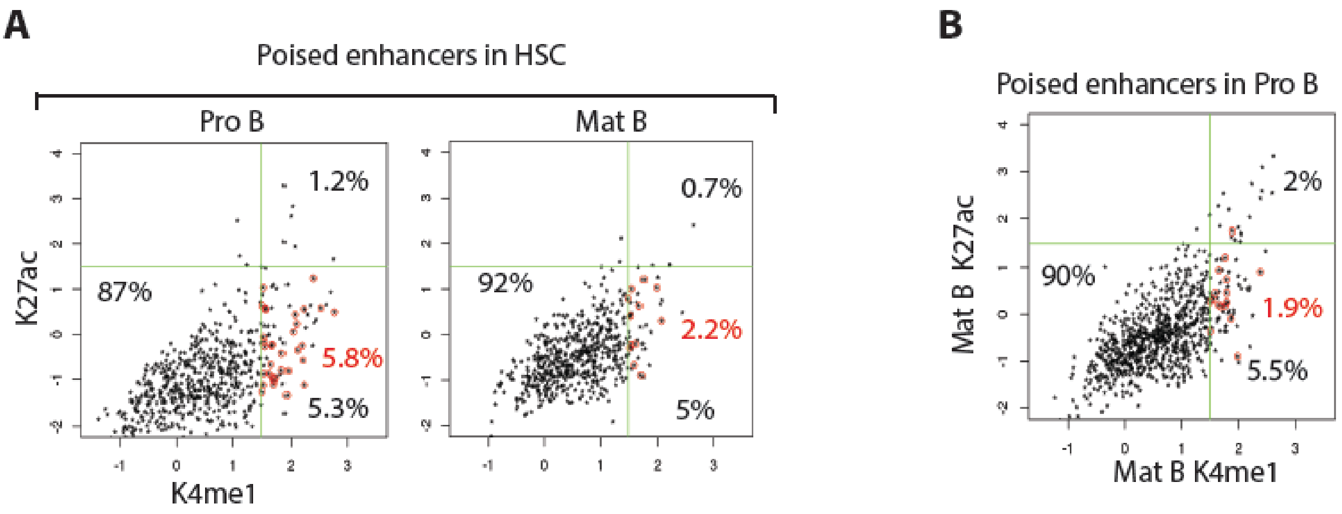

Supplementary Fig. 4: Behavior of poised enhancers during differentiation.

A. The chromatin state of enhancers poised in HSCs was investigated in Pro B and mature B cells. H3K4me1 and H3K27ac enrichment in the indicated cell types were calculated at genomic coordinates corresponding to poised enhancers in HSCs. Green lines indicate cut-offs used to select enriched regions for each signal. The proportions of the different population are indicated in the scatter plots. Poised enhancers in the indicated stages are marked by red circles and their proportions are indicated in red.

B. Similar to A, the chromatin state of enhancers poised in Pro B cells was investigated in mature B cells.

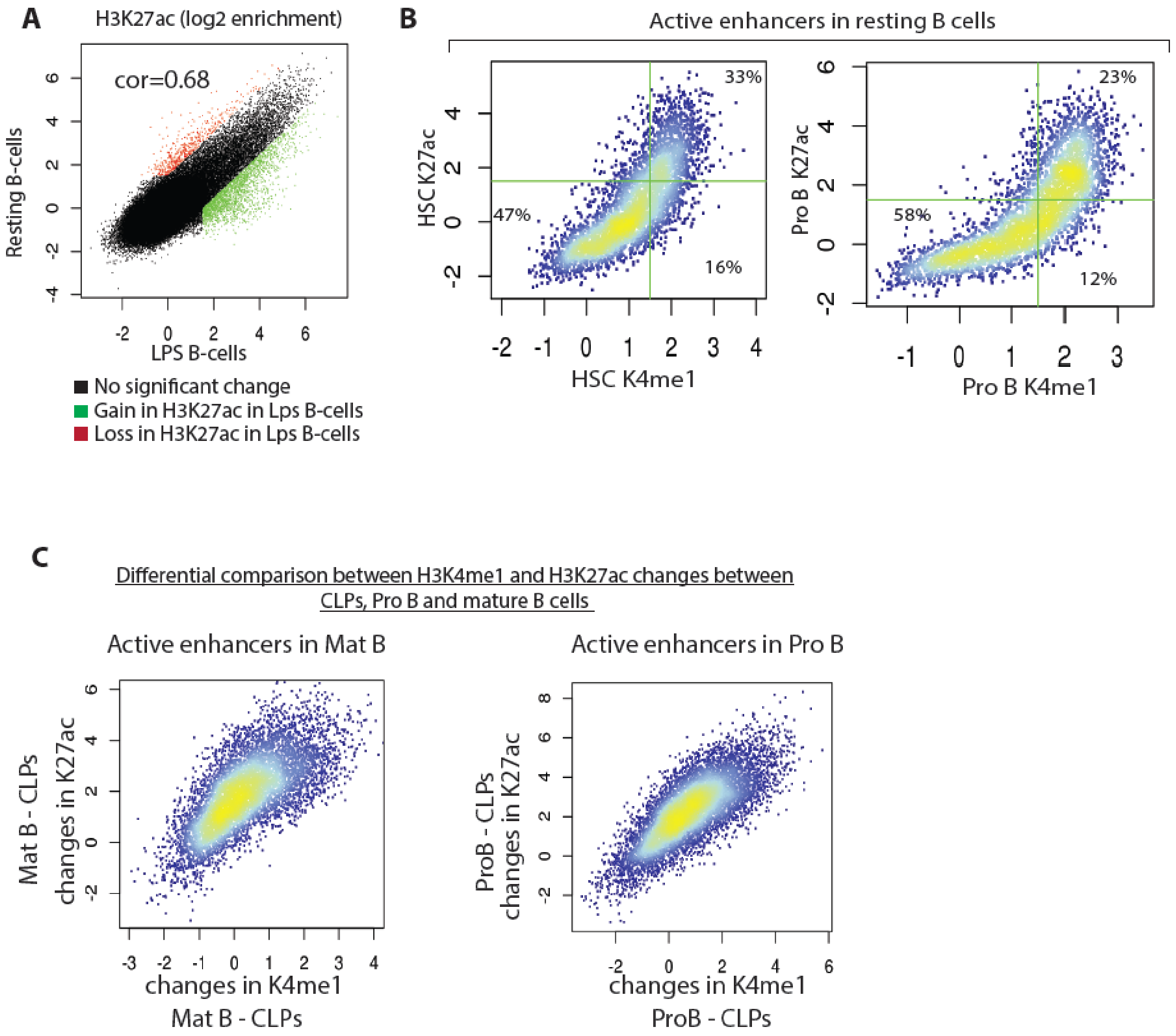

Supplementary Fig. 5: Comparison of active enhancers in resting and LPS-activated B cells.

A. H3K27ac enrichment (over input) in 1kb sliding windows across the genome for LPS-stimulated and resting B cells. Loci with no change in H3K27ac signal between the two conditions are colored in black, loci showing an increase in H3K27ac signal in LPS stimulated cells compared to resting cells are colored in green and loci with a decreased H3K27ac enrichment are colored in red. A 1.5 fold cut-off was used to select differentially enriched loci.

B. The chromatin state of active enhancers in resting B cells (identified based on H3K27ac signal) was investigated in HSCs (left panel) and Pro B cells (right panel).

C. Scatter plots for changes in H3K27ac and H3K4me1 between mature B cells and CLPs (left panel) and between Pro B cells and CLPs (right panel).

A Distribution of expression values (log2 scale)

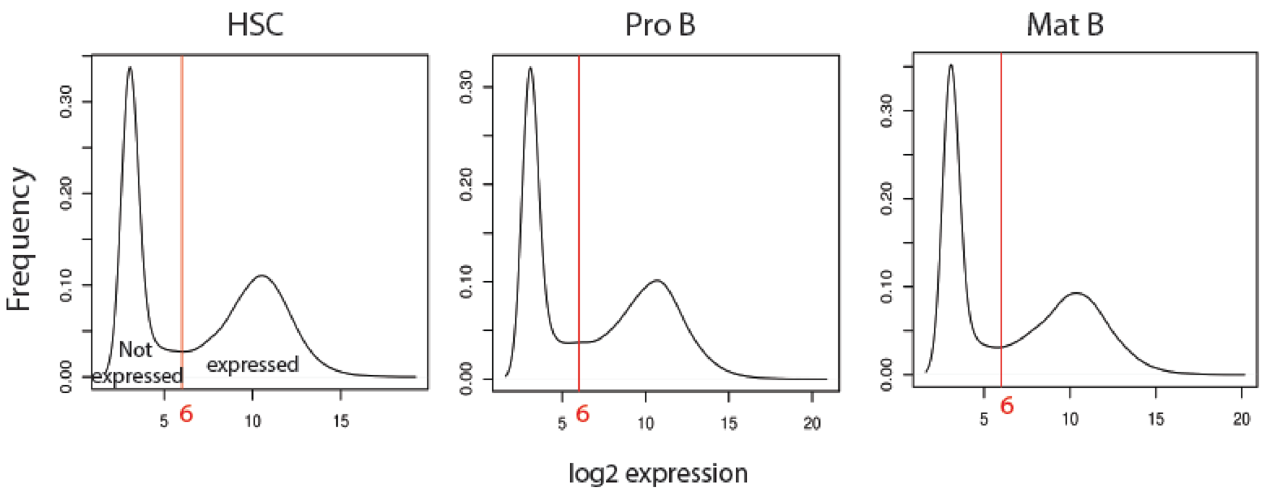

Supplementary Fig. 6: Gene classification according to expression level.

A. Histograms showing the distribution of the log2 expression levels as determined by RNA-sequencing experiments (see Material and Methods) in HSCs, Pro B and mature B cells. The cut-off to classify a gene as expressed was set to 6 (red horizontal line).

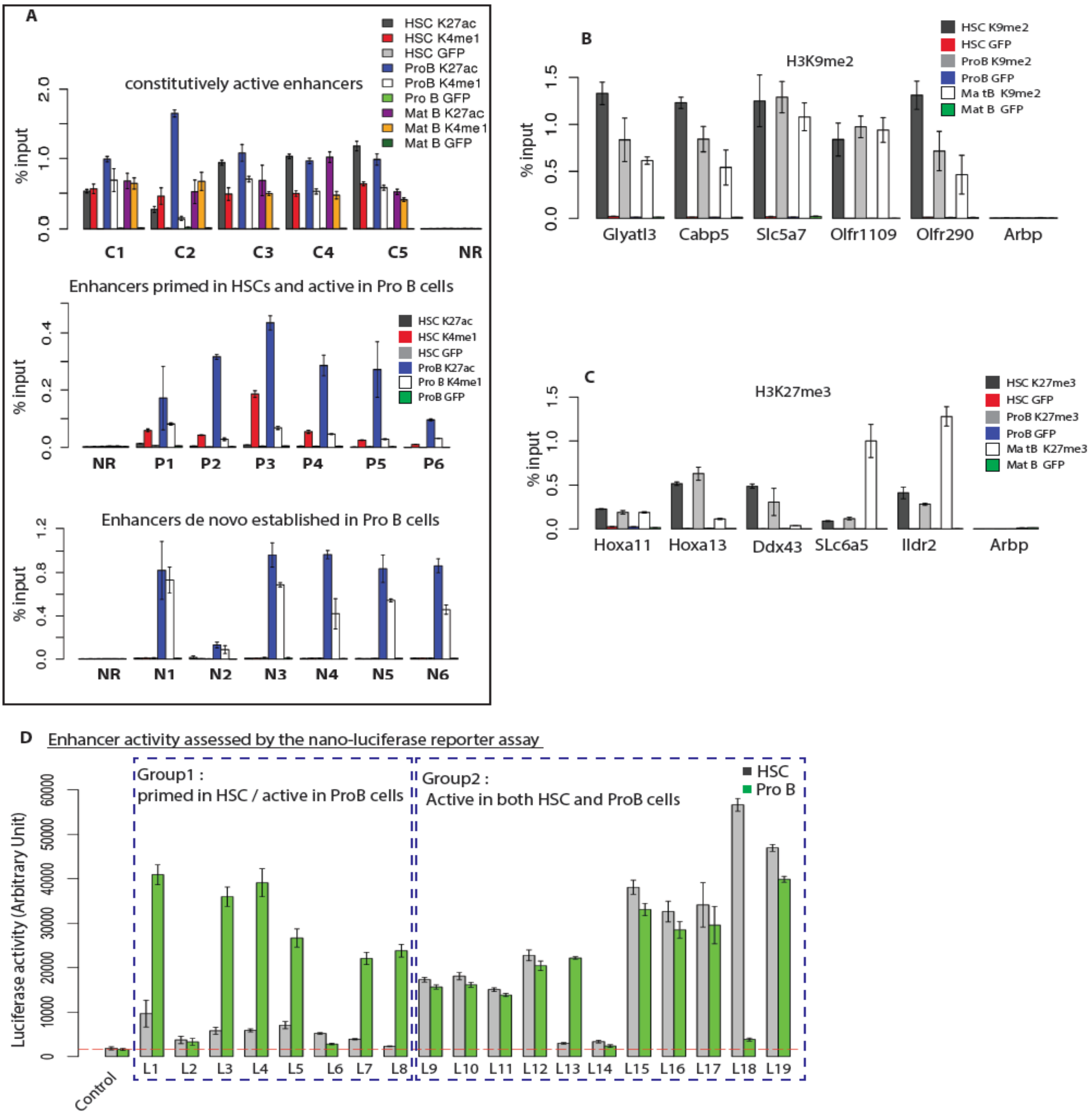

Supplementary Fig. 7: ChIP qPCR and reporter assays validation of ChIP sequencing data.

A. A subset of enhancers from different categories were validated by independent ChIP qPCR experiments; the first panel represents constitutively active enhancers (C1 to C5), the second panel represents enhancers primed in HSCs and active in Pro B cells (P1 to P6) and the third panel represents enhancers de novo generated in Pro B cells (N1 to N6). The testis specific locus Protamine1 was used as a negative control. Genomic Coordinates of these loci are provided into supplementary Table 3.

B and C. A subset of genes enriched in H3K9me2 (B) and H3K27me3 (C) marks were investigated by ChIP qPCR; the highly expressed ribosomal protein gene *Arbp* was used as a negative control.

D. Luciferase activity for a subset of primed and active enhancers in HSCs and Pro B cells. The coordinates of the loci used in this figure are given in the table S1.

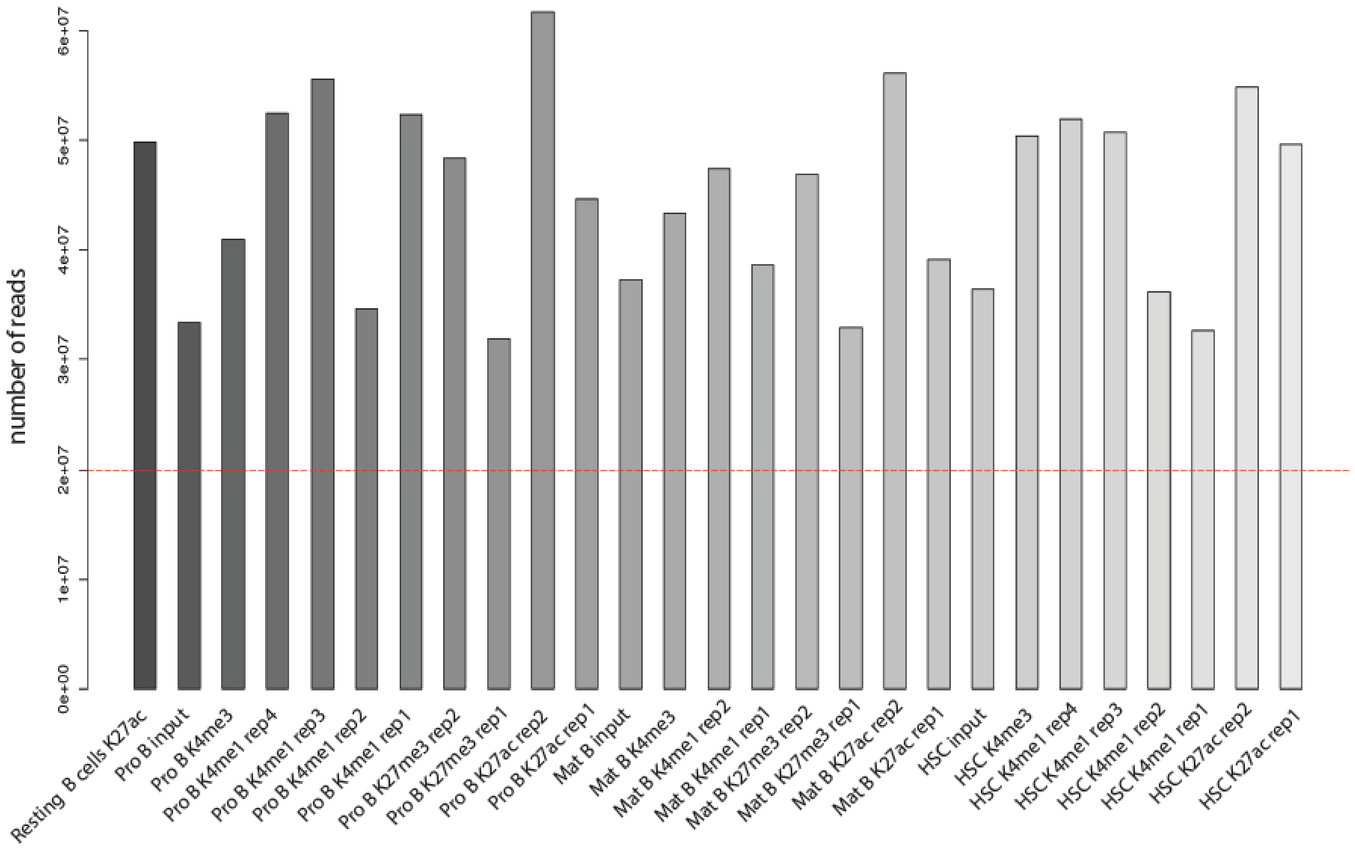

Supplementary Fig. 8: Barplot representing the number of reads for each ChIP-sequencing experiment.

Supplementary Tables

Supplementary Table 1: Coordinates of enhancers used in reporter assays (Figure 1D, Supplementary Fig. 7D).

Locus Name	Coordinates
L1	chr11: 100591638-100592913
L2	chr2:118324225-118325286
L3	chr19: 47612048- 47613191
L4	chr1: 129108567-129109172
L5	chr2: 90371166-90372676
L6	chr1:130571787-130572987
L7	chr7:133559137-133561153
L8	chr10:94605090-94606684
L9	chr2:144056107-144057375
L10	chr11: 100591638-100592913
L11	chr2:118324225-118325286
L12	chr9:45081937-45082864
L13	chr4:9601957-9602736
L14	chr4: 9603272- 9603860
L15	chr17:47658318-47659541
L16	chr9:13897323-13898563
L17	chr7:73378119-73379155
L18	chr16:92620823-92621911
L19	chr2:90921161-90925642

Supplementary Table 2: Public data sets used in this study.

GEO sample ID	Sample
GSM1441284	H3K27Ac_CLP
GSM1441300	H3K4me1_CLP
GSM769031	Spleen H3K4me1
GSM1000138	Spleen H3K27ac
GSM769036	Spleen H3K4me3
GSM769037	Spleen input
GSM918705	Thymus input
GSM1000101	Thymus H3K4me3
GSM1000102	Thymus H3K4me1
GSM1000103	Thymus H3K27ac
GSM1164635	HSC H3K27me3 rep1
GSM1164636	HSC H3K27me3 rep2

Supplementary Table 3: Coordinates and qPCR primers for enhancers verified by ChIP-qPCR in Supplementary Fig. 7A.

Locus Name	Coordinates	fw primers	rev primers
C1	chr2:144073306-144073986	ttcagctggacacacacaca	ggctgtcctggaactctgtc
C2	chr13:103482392-103483524	gtggtgtccattgtgagtgc	atggcctactgcatttcctg
C3	chr9:45081937-45082864	ctggggaacagatttgcatt	aattctccctttccctccaa
C4	chr17:47658919-47660312	agttggctcaatgggaacac	aaccactgttcaggggtcag
C5	chr2:90921161-90925642	aggtcatcaagttccgcaga	ctgagtgggtgggttctgat
P1	chr18:35974566-35975392	gattgcccagagtgtggagt	gcccacctggatctaactca
P2	chr11:100589876-100591397	gcaggggagtcagcagtaag	aagactgttctccggcttca
P3	chr2:118324225-118325286	ggaagctgtgtggaaagctc	gtctcctaaggcagcaggtg
P4	chr19:47612048-47613191	tcagcctccctcctgtagaa	atatcgatcggccttgaatg
P5	chr1:129108567-129109172	acgcagtggaaggagaagaa	agctgatgatcccatgatcc
P6	chr2:90371166-90372676	accatctcggggaaaactct	gctggatggtggcagtaaat
N1	chr7:108617991-108619209	tgaggtggcaatgaaatgaa	caggtcccacacagtcattg
N2	chr13:73847304-73848770	gccagccagctgtttcttac	gctaaggatcgcagtcaagc
N3	chr11:45351843-45353015	ttctgcaacatcctcactgc	catgaaagcggagacacaga
N4	chr5:137154497-137155688	gtaaaagctgtgggctgagg	acacccacaggtgagactcc
N5	chr5:83382743-83384187	gaatgccccacgttaagaga	atgttctcccagcaaaccac
N6	chr4:44683297-44688461	agcgagttgtgaaggtcaga	catcacgcagcagaactctc

Supplementary Table 4: Primers of genes verified by qPCR in Supplementary Fig. 7B and C.

Gene name	fw primers	rev primers
Glyatl3	ctgggtttcccttctcatca	accccgagactgacctacct
Cabp5	aggaggagttggcaaaggat	gaaactgcattggagcaggt
Slc5a7	cccccaaaaacatcaaatct	tgcatataatggttccaaaaca
Olfr1109	tccccagttacccacaaaaa	catggacccagggaatcata
Olfr290	tggggtcattactcccatgt	accaaaagacacaggccaac
Arbp (rplp0)	gtcgatggaaccagccaata	cctcccacaacaaaacaacc
Hoxa11	aggagccttcctttcagctc	ggccctttcaaagtctttcc
Hoxa13	ccccttccatgttcttgttg	cttcacttcttgggggcttt
Ddx43	cattagcccgtcatgaacct	gctagtgtctggctgggaac
Slc6a5	cattagcccgtcatgaacct	gctagtgtctggctgggaac
Ildr2	tcttatcgtgccagctgatg	acgaaggtggagtggaacac
